# Improvement of Overall Survival Using TKIs as Salvage Therapy in Advanced Thyroid Carcinoma: Real-Life Data on a Single Center Experience

**DOI:** 10.3390/jcm10030384

**Published:** 2021-01-20

**Authors:** Lucia Brilli, Cristina Dalmiglio, Tania Pilli, Filomena Barbato, Fabio Maino, Marco Capezzone, Alessandra Cartocci, Maria Grazia Castagna

**Affiliations:** 1 Department of Medical, Surgical and Neurological Sciences, University Hospital of Siena, 53100 Siena, Italy; lucia.brilli@ao-siena.toscana.it (L.B.); cristina.dalmiglio@student.unisi.it (C.D.); t.pilli.e@ao-siena.toscana.it (T.P.); filomena.brb@hotmail.it (F.B.); fabio.maino@ao-siena.toscana.it (F.M.); m.capezzone@ao-siena.toscana.it (M.C.); 2 Department of Medical Biotechnologies, University of Siena, 53100 Siena, Italy; alessandra.cartocci@dbm.unisi.it

**Keywords:** tyrosine kinase inhibitors, thyroid cancer, salvage therapy

## Abstract

*Background:* Tyrosine kinase inhibitors (TKIs) have improved progression-free survival in patients with advanced thyroid cancer. So far, few studies have investigated the efficacy of TKIs in a second-line setting. The purpose of our study was to explore the salvage therapy efficacy in patients with advanced thyroid cancer. *Methods:* We retrospectively evaluated 63 patients with progressive advanced thyroid carcinoma treated with TKIs divided into a Study group (23 patients) treated with salvage therapy, and a Control group (40 patients) treated with only one TKI. *Results:* Similar clinical benefits (stable disease + partial response) and progression free survival between the first and the second line TKI were observed in the Study group (*p* > 0.99 and *p* = 0.5, respectively). Median overall survival (OS) was 67.7 months in the Study group and 22.6 months in the Control group (HR 2.46; 95% CI 1.34–4.52, *p* = 0.004). After stratifying the whole population by age (<65 and ≥65 years), OS was significantly different (*p* < 0.001) with the best survival curve in younger patients, treated with salvage therapy and the worst in older subjects, treated with only one TKI. *Conclusions:* Salvage therapy showed a significant improvement of OS in patients with advanced thyroid cancer who experienced disease progression during prior TKI therapies.

## 1. Introduction

Approximately 5–10% of patients with differentiated thyroid cancer (DTC) develop metastatic disease over time and among them two-thirds have iodine refractory disease [1]. In this subgroup of DTC patients, the 10-year survival rate is less than 20% [2]. In medullary thyroid carcinomas (MTCs) distant metastases are present at diagnosis in 7–23% of patients and represent the main cause of cancer-related death with a 10-year survival rate disease ≤40% compared to a 10-year overall survival of 75% observed in unselected MTC patients [3,4]. Four multikinase inhibitors (vandetanib, cabozantinib, sorafenib and lenvatinib), all targeting the vascular endothelial growth factor receptor (VEGFR), have obtained regulatory approval for advanced thyroid cancer as a result of significant improvement in progression free survival (PFS) demonstrated in phase III trials [5,6,7,8]. Several other related agents have been tested in clinical trials or used off-label as first- and second-line treatments for selected patients [9,10]. Despite the evidence of a reasonable efficacy, most patients could no longer respond to tyrosine kinase inhibitors (TKIs) due to drug resistance, and many patients were forced to discontinue the drug prematurely because of the toxic side effects. In these cases, salvage therapy with another TKI is suggested. In other types of human cancers, the efficacy of sequential treatment with TKIs has been demonstrated, even if the ideal sequence of targeted agents requires further elucidation [11,12,13]. The first available data on utility of second-line TKIs therapy in advanced thyroid carcinoma are provided by monocentric studies or randomized international multicentric trials [5,6,8,14,15,16,17]. No significant differences were observed in terms of PFS and/or response rate defined according to the Response Evaluation Criteria in Solid Tumors (RECIST), among patients previously treated or not with others TKI. To date, only a few studies have specifically investigated the efficacy of second-line TKIs in advanced thyroid carcinoma [18,19,20,21,22,23].

In our retrospective study, we try to evaluate the efficacy of salvage therapy in patients with advanced thyroid cancer, including DTC, poorly differentiated thyroid cancer (PDTC) and MTC, who experienced disease progression after first-line TKI treatment.

The primary objective of this study was to evaluate the efficacy of TKI, when used as first- or second-line, in terms of best overall response (BOR) and progression free survival (PFS). The secondary objective was to compare the overall survival (OS) among patients treated with only one TKI and patients treated with salvage therapy after failure of the first TKI treatment.

## 2. Materials and Methods

### 2.1. Study Population

We retrospectively evaluated 63 patients with progressive advanced thyroid carcinoma who received first-line treatment with TKI from November 2004 to September 2017 at our institution. The extended follow-up continued to January 2020. The patient data collected included age, sex, histological findings, site of distant metastases, information about treatment with TKIs (time lapse between diagnosis and treatment start, duration of treatment, reason for discontinuation), information about any salvage treatments received, tumor response, and follow-up time or date of death. The patients were divided into two groups: the Study group included patients treated with sequential TKIs after first TKI failure (salvage therapy) and the Control group was composed of patients treated with only one TKI.

### 2.2. Assessments and Definitions

Patients were submitted at baseline and every 8–12 weeks to radiological evaluation by total body computed tomography (CT) or magnetic resonance imaging (MRI). The response to therapy was evaluated according to RECIST criteria v.1.1 [24]. PFS was defined as the time elapsed from TKI administration to the first evidence of tumor progression documented by CT or MRI examination according to RECIST criteria v 1.1 or until death. Best overall response (BOR) was defined as the best response recorded from the start of the treatment until disease progression/recurrence. We considered a clinical benefit when BOR was partial response (PR) or stable disease (SD). The overall survival (OS) was calculated from the start date of the TKI treatment to the time of death from any cause.

### 2.3. Statistical Analysis

Quantitative variables were presented as the mean ±SD, and with median when normality cannot be statistically established. The Anderson–Darling test was used to assess normality of variables’ distribution. The independent *t*-test or the Mann–Whitney test were performed for normal or non-normal variables, respectively. Qualitative variables were presented as absolute frequencies and percentages and to evaluate significant differences in data frequency we analyzed 2 × 2 contingency tables by the Fisher exact test. Tables with size larger than 2 × 2 were examined by the Chi-squared test or by numerical approximation of the Fisher exact test, when all expected frequencies were greater than 5 or not, respectively. The progression free survival and the overall survival were evaluated by Kaplan–Meier curves and to evaluate differences between two or more survival curves log-rank test was performed. Stepwise Cox regression was used to examine factors contributing to overall survival and the hazard ratio (HR) with their 95% confidence interval (CI) are estimated. A *p*-value <0.05 was considered statistically significant. Statistical analysis was performed using the software StatView for Windows version 5.0.1 (SAS Institute, Cary, NC, USA) and the SPSS Statistics version 22.0.

## 3. Results

### 3.1. Study Population

Clinical and pathological features of study population are reported in Table 1. Forty-two patients (66.7%) had progressive advanced RAI-refractory DTC, 5 patients (7.9%) had PDTC while 16 patients (25.4%) had progressive metastatic or locally advanced MTC. At cancer diagnosis patients (*n* = 63) had a mean age of 56.4 ± 17 years and 52.4% of them were males. Mean age at the time of TKI treatment was 63.5 ± 15.2 years and the time-lapse between the diagnosis of thyroid cancer or the appearance of distant metastases and the start of therapy with TKI was 7 ± 6.81 years and 3.6 ± 3.3 years, respectively. Fifty-seven percent of the entire cohort had three or more metastatic sites. Bone metastases were present in 29/63 patients (46%), lung metastases in 43/63 (68.2%) and the target lesion sum at baseline was 84.83 ± 55.2 mm.

Patients were divided into two groups: the Study group consisted of 23 patients treated with sequential TKIs after first TKI’s failure (salvage therapy) and the Control group was composed by 40 patients treated with only one TKI (Table 1). Among control group, the majority of patients did not receive a second line treatment because it was not currently available. Fourteen/40 (35%) patients in the control group and 14/23 (60.8%) in the study group received a 1st line TKI and 8/23 (34.8%) patients in the study group received a 2nd line TKI within a clinical trial.

The patients treated with more than one TKI (Study group, *n* = 23) and those treated with only one TKI (Control group, *n* = 40) were not statistically different except for age, in details the patients of Study group were younger both at the time of cancer diagnosis (53 vs. 61 years, *p* = 0.004) and at the time of starting TKI (59 vs. 68 years, *p* = 0.007) (Table 1).

### 3.2. TKIs Treatment in the Entire Population

TKIs used as first line therapy did not differ between the Study group and the Control group (*p* = 0.22). Specifically, in the Study group, sorafenib was the most frequent TKI used (43.5% of subjects), followed by vandetanib (26.1%), motesanib (13%), sunitinib (8.7%) and lenvatinib (8.7%). Similarly, in the Control group the majority of patients were treated with sorafenib (52.5%), followed by vandetanib (25%), lenvatinib (17.5%) and motesanib (5%), while no patients were treated with sunitinib.

In the Study group, a second TKI treatment was started for disease progression in 19/23 (82.6%) patients and for drug-related adverse events in 4/23 (17.4%) cases. The second line therapy included lenvatinib in 8/23 (34.8%) patients, sorafenib in 5/23 (21.7%), sunitinib in 6/23 (26%), cabozantinib in 2/23 (8.7%), pazopanib and vandetanib one patient each (4.3%).

### 3.3. Efficacy Analysis of TKIs Treatment in the Study Group (n = 23)

**Figure 1:**
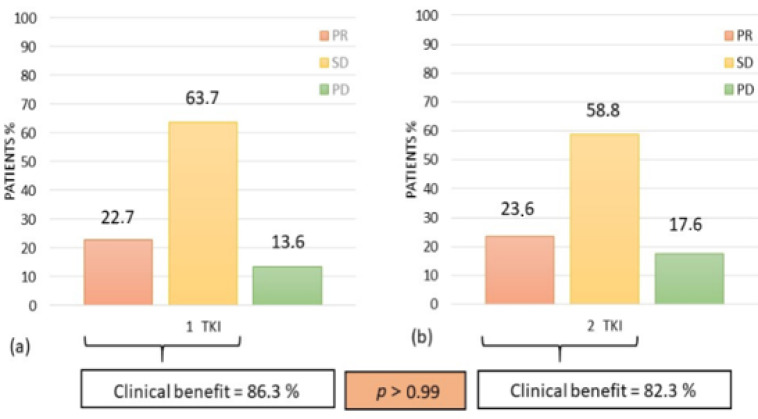
Best overall response and clinical benefit in the Study group: (**a**) with the first TKI (**b**) with the second TKI. PR: partial response; SD: stable disease; PD: progressive disease.

In the Study group, the BOR with the first TKI (available in 22/23 patients (95.6%)) was PR in 5/22 (22.7%), SD in 14/22 (63.7%), and PD in 3/22 (13.6%) patients. For the second TKI the BOR was analyzed only in patients who stopped the first TKI for disease progression (17/22, 77.3%), excluding from the statistical analysis patients who experienced toxicity that led to discontinuation of first TKI (*n* = 5). Best overall response with the second TKI was PR in 4/17(23.6%), SD in 10/17 (58.8%) and PD in 3/17 (17.6%) patients (Figure 1).

**Figure 2:**
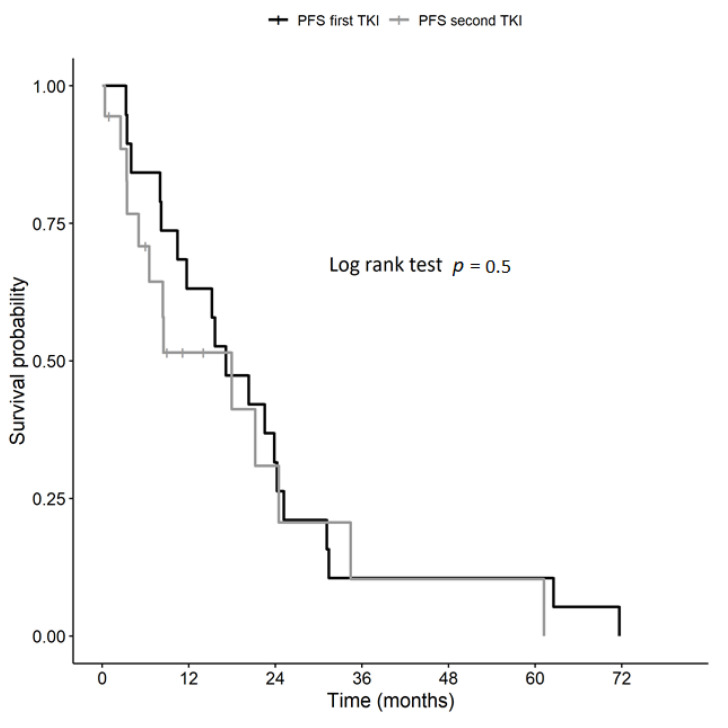
Kaplan–Meier curve of progression-free survival (PFS) in the Study group (>1 TKI).

Overall, a clinical benefit was observed in 19/22 (86.3%) of patients during treatment with the first TKI and in 14/17 (82.3%) of patients according to second TKI (salvage therapy) without a significant difference between the first and second TKI (*p* > 0.99). These results were obtained after a median of 6.5 ± 6.4 months (range 1.6–24.8 months) with the first TKI and after a median of 3.78 ± 10.0 months (range 1.26–40.40 months) with the second TKI. In a subgroup of patients who experienced a clinical benefit (with a first-line TKI in 21/22 patients (95.4%) and with a second-line TKI in 15/17 patients (88.2%)), we evaluated the median percentage reduction of the sum of target lesions. It was 18.6% (range −58.9%–+14.5%) and 16.7% (range −44.8%–+10.8%) with the first and the second TKI, respectively without significant difference (*p* = 0.8). The median PFS was 17.1 months (mean 21.6 ± 4.1 months, range: 3.3–71.7 months) with first-line TKI, not significantly different (Log rank test *p* = 0.5) from that observed with salvage therapy (median 18 months; mean 18.8 ± 4.9 months, range: 0.36–61.2 months) in the Study group (Figure 2).

### 3.4. Overall Survival in the Study (n = 23) and in the Control group (n = 40)

**Figure 3:**
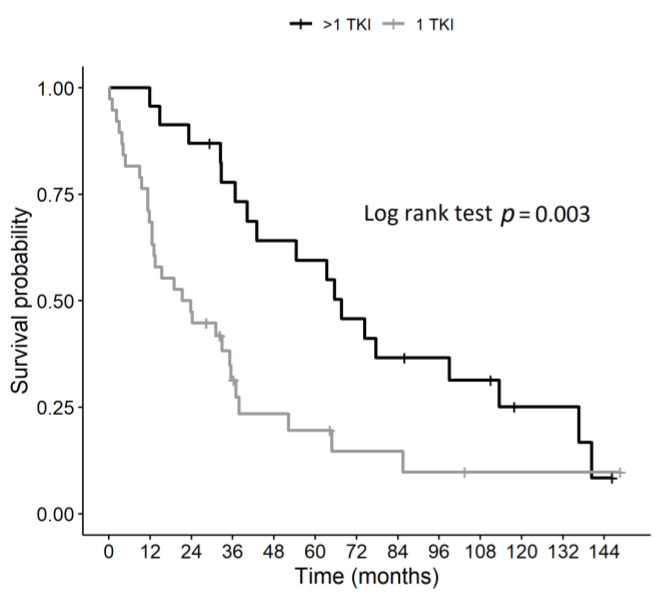
Kaplan–Meier curve of overall survival in Study (>1 TKI) and Control group (1 TKI).

As shown in Figure 3, the OS of the Control group was significantly lower than the Study group (Log rank test *p* = 0.003). The median OS in the Study group (the salvage therapy group) was 67.7 months (mean 76.8 ± 9.6 months, range 11.8–146.3 months), which was longer than that observed in the Control group (22.6 months, mean 37.4 ± 7.7 months, range 0.26–148.7) (HR 2.46; 95% CI 1.34–4.52, *p* = 0.004). Specifically, the 3- and 5-year survival rates were 78% (CI: 62%–97%) and 60% (95% CI: 42%–84%) in the Study group and 31% (19%–52%) and 20% (CI: 9%–41%) in the Control group.

### 3.5. Overall Survival Based on the Age of TKI Treatment Start

**Figure 4:**
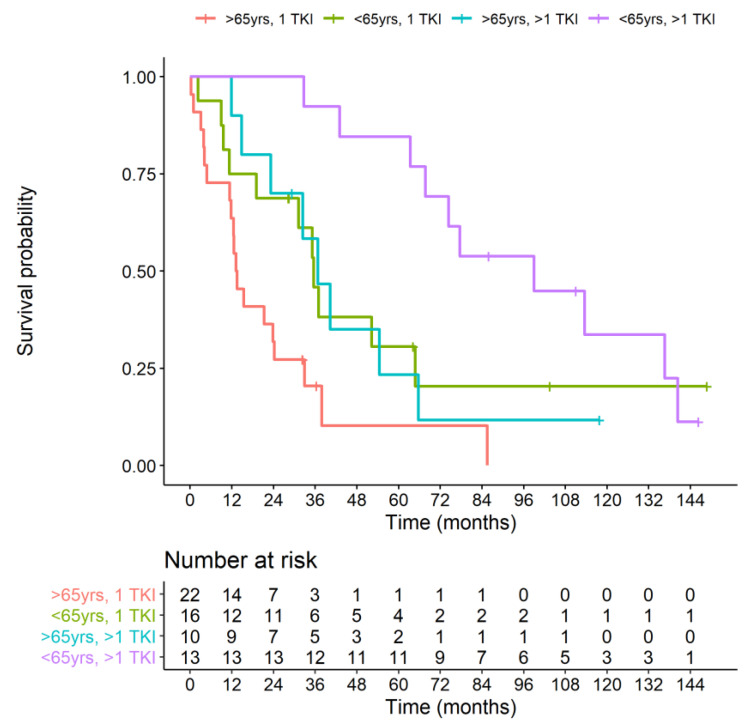
Kaplan–Meier curve of overall survival of Study (>1 TKI) and Control group (1 TKI) stratified by age (> or <65 years).

Both groups were also stratified according to the age of TKI treatment start (<65 and ≥65 years). Kaplan–Meier curve analysis showed an OS significantly different between the four groups (*p* < 0.001). Of note, the best survival curve was obtained in younger patients (<65 years) treated with salvage therapy while the worst in older subjects (>65 years) treated with only one TKI (Figure 4).

Salvage therapy was able to significantly improve OS in older patients (median OS: 35.5 months vs. 13.3 months (HR 2.36, 95% CI, 1.00–5.56, *p* = 0.04)) while a trend toward significance was observed in younger patients (median survival 99 months vs. 36.7 months in younger patients (HR 2.24, 95% CI, 0.92–5.45, *p* = 0.07)). Moreover, salvage therapy was more effective in younger patients when compared with older subjects (median survival 99 months in patients <65 years and 35.5 months in patients >65 years (HR 2.88, 95% CI, 1.55–5.32 *p* = 0.0008).

### 3.6. Prognostic Factors Associated with Survival Benefit

Stepwise Cox regression was used to examine factors contributing to overall survival. Variables included in the analysis were gender, time-lapses between metastasis appearance and TKI treatment, number of sites of distant metastases (>2 vs. ≤2), presence of bone metastases, sum of the target lesions diameters, age at cancer diagnosis and at the beginning of the first TKI, number of TKI line of treatment (1 TKI vs. >1 TKI) and histology (DTC or PDTC vs. MTC).

We found that more than two sites of distant metastasis (HR 2.82; 95% CI, 1.43–5.57, *p* = 0.003), a greater sum of the target lesions diameters (HR 1.01; 95% CI, 1.00–1.02, *p* = 0.012), older age at the beginning of the first TKI (HR 1.07; 95% CI, 1.05–1.1, *p* < 0.001) and treatment with only one TKI (HR 3.23; 95% CI, 1.5–6.60, *p* = 0.001), negatively affected OS.

## 4. Discussion

Since resistance to TKIs is frequent, salvage therapy, after failure with first-line TKI treatment, is routinely used in the clinical practice. Moreover approximately 20% of patients permanently discontinue TKIs for drug-related adverse events [5,6,7,8]. To date, salvage therapy in advanced thyroid carcinoma was evaluated only in few studies and its efficacy was not well defined [18,19,20,21,22,23]. Our results suggest that treatment with TKIs can be effective also in the second-line setting in thyroid cancer patients. Indeed, the rate of clinical benefits and PFS that occurred in patients treated with the first TKI was maintained with the second-line treatment. So far, similar results have been obtained in only two studies that investigated the effect of salvage therapy in patients with advanced thyroid carcinoma [19,21]. In particular, Dadu et al. [19] evaluated the efficacy of different TKIs as salvage therapy, after first-line treatment failure (sorafenib) in a cohort of 25 patients with metastatic, radioactive iodine (RAI)-refractory DTC. The median PFS with the second-line therapy was 11.4 months, similar to that observed with sorafenib (7.4 months) [19]. In a retrospective study including 24 patients with advanced DTC treated with sorafenib or sunitinib, PFS in the first-line setting was similar to that observed in the second-line (7.0 vs. 6.7 months, respectively), even though no PR was observed in the salvage therapy group [21]. On the contrary, other authors reported a lower PFS during salvage therapy compared to that observed in the first-line setting [22,25].

Survival data of patients with advanced thyroid cancer treated with TKI are limited. In phase III trials no OS benefit was found in patients treated with TKI compared to placebo [5,6,7,8]. Moreover, to date only two studies demonstrated a significant improvement of OS in patients treated with sequential TKI compared to those treated with TKI in monotherapy [19,23]. An important finding of our study was the longer OS observed in patients undergone salvage therapy. The median OS was 67.7 months for those who received salvage therapy compared to 22.6 months in those treated with one TKI (*p* = 0.004). Our results are consistent with those reported by Dadu et al., who evaluated salvage therapy in 60 patients with DTC after discontinuation of first-line sorafenib [19], but unlike to our study, they included in the analysis not only patients with progressive disease, but also subjects who discontinued the first-line TKI because of adverse events.

Through a stepwise Cox regression, we found that more than two sites of distant metastases (HR 2.82), a greater sum of the target lesions diameters (HR 1.01), older age at the beginning of the first TKI (HR 1.07) and treatment with only one TKI (HR 3.23), negatively affected OS. The clinical impact of age and salvage therapy on the survival was also confirmed by Kaplan–Meier analysis. Accordingly, to the age at the time of the first TKI, salvage therapy was able to improve OS in both younger and older patients, although in patients <65 years only a trend toward significance was observed. Moreover, salvage therapy was more effective in younger patients (median survival 99 months) when compared with older subjects (median survival 35.5 months, *p* = 0.0008). It is known that age represents an important prognostic factor for thyroid cancer mortality, that increases with each decade of life beyond age 40, showing a marked increase of mortality in patients aged >60 years [26,27]. Nevertheless, the positive impact of younger age on OS observed in our study, could also be linked to a better tolerability toward the drugs and a lower frequency of drug-related adverse events in young patients, who therefore may require less frequently drug withdrawal or dose reduction [28]. While age plays a pivotal role, it should be pointed out that salvage therapy has a significant positive impact in OS also in older patients. These results suggest that not only younger age but also salvage therapy is able to improve overall survival in patients with advanced thyroid cancer.

To our knowledge this is the first study in which the efficacy of salvage therapy, according to the age at the first TKI start, was evaluated. The role of age in advanced thyroid cancer patients treated with lenvatinib was investigated in a post-hoc analysis of the SELECT trial, in which a significant improvement of OS was observed only in the subgroup of patients older than 65 years. However, conversely to our study were that lenvatinib was used as a second-line treatment only in 25% of patients and its effect on OS, as salvage therapy, was not evaluated. The effect of age on lenvatinib efficacy was also investigated in a recent Italian real-world study conducted in an unselected cohort of advanced DTC patients. Lenvatinib was used as second line treatment in the majority of patients, but as previously, the authors did not perform any comparison between the treatment lines [29].

Some limitations of our study are intrinsic to its retrospective design. Because advanced thyroid cancer is rare and TKIs are quite recent treatment option, the subgroup of patients in our study population who received salvage therapy was small, they received a variety of different drugs and patients with different histotypes of thyroid cancer were included. Due to all these limitations, we were not able to perform and additional analysis according to the drug administered and the histological features. On the other hand, the study has several strengths including a similar therapeutic approach and follow-up at the same institution and evidence of progressive disease before the salvage start in all patients. In addition, at our knowledge, this is the first study that has validated the role of salvage therapy in OS according to patient age.

The results of our study confirm the efficacy of salvage therapy in advanced thyroid carcinoma in terms of PFS and OS. The mechanisms underlying the clinical benefits in the second-line setting are not fully established, but they could be related to the ability of TKIs to act on different tyrosine-kinase receptors or act in the same receptor, with a different affinity, explaining the different efficacy of the TKIs and the onset and magnitude of the adverse events.

Moreover, since the patients become resistant to treatment with TKIs over time, novel therapeutic approaches are needed in addition to the standard treatment. Recent studies showed that ligand of programmed cell death protein 1 (PD-L1) expression was detected in thyroid cancer, with low level in DTC and more diffuse expression in PDTC and anaplastic cancer (ATC), in which the positive rate reaches up to 70–90% [30,31]. Combination therapies that target both the tumor and the immune response are currently under investigation in follicular-derived advanced thyroid carcinoma with promising results [31], and this kind of approach could contribute to a better prognosis in patients with advanced thyroid cancer.

In conclusion our study shows that salvage therapy in patients with advanced thyroid carcinoma is effective and, if available, should always be offered to patients who develop disease progression under TKI treatment or who cannot continue therapy due to drug-related adverse events.

## Figures and Tables

**Table 1:** Patient characteristics for the whole population (*n* = 63) and comparison between Study group (*n* = 23) and Control group (*n* = 40).

	All Patients	Study Group	Control Group	*p* Value
		(>1 TKI)	(1 TKI)	
Patients (*n*)	63	23	40	
Male Sex *n* (%)	33 (52.4%)	12 (52.17%)	21 (52.5%)	0.99
Histology *n* (%)				0.6
DTC	42 (66.7%)	15 (65.2%)	27 (67.5%)	
PDTC	5 (7.9%)	1 (4.3%)	4 (10%)	
MTC	16 (25.4%)	7 (30.5%)	9 (22.5%)	
Age at cancer diagnosis (years)				0.0046
Mean (range)	56.43 (23.20–85.35)	48.56 (23.21–78.83)	60.96 (29.88–85.35)	
Median	57.56	53.75	61.07	
Age at the time of TKI treatment (years)				0.0073
Mean (range)	63.51 (25.57–88.01)	56.85 (25.57–80.93)	67.35 (32.67–88.01)	
Median	65.51	59.18	68.32	
Time-lapse between cancer diagnosis and TKI treatment (years)				0.29
Mean (range)	7.08 (0.09–40.05)	8.28 (0.68–40.05)	6.39 (0.09–25.09)	
Median	5.68	5.9	4.94	
Time-lapse between appearance of metastases and TKI treatment (yrs)				0.98
Mean (range)	3.59 (0–14.23)	3.6 (0.05–9.79)	3.58 (0–14.23)	
Median	2.75	3.08	2.73	
Numbers of anatomical site involved *n* (%)				0.51
1	9 (14%)	3 (13.5%)	6 (15%)	
2	18 (29%)	4 (17%)	14 (35%)	
3	22 (35%)	9 (39%)	13 (32.5%)	
4	7 (11%)	4 (17%)	3 (7.5%)	
>4	7 (11%)	3 (13.5%)	4 (10%)	
Bone metastases *n* (%)	29 (46%)	11 (48.8%)	18 (45%)	0.82
Lung metastases *n* (%)	43 (68.2%)	18 (78.2%)	25 (62.5%)	0.27
Sum of the target lesion diameters (mm) at baseline *				0.25
Mean (range)	84.83 (17–275)	74.32 (21–171)	91.84 (17–275)	
Median	65	64.65	65	
ECOG performance status *n* (%) **				0.8
0–1	57 (93%)	22 (95.7%)	35 (92.1%)	
2	4 (6.5%)	1 (4.3%)	3 (7.9%)	

TKI, tyrosine kinase inhibitor; DTC, differentiated thyroid cancer; PDTC, poorly differentiated thyroid cancer; MTC, medullary thyroid cancer; ECOG, Eastern Cooperative Oncology Group. * evaluable in 22/23 patients in the Study group and in 33/40 patients in the Control group. ** evaluable in 23/23 patients in the Study group and in 38/40 patients in the Control group.

## Data Availability

Data is contained within the article.

## References

[1] Durante C., Haddy N., Baudin E., Leboulleux S., Hartl D., Travagli J.P., Caillou B., Ricard M., Lumbroso J.D., Vathaire F.D. (2006). Long-term outcome of 444 patients with distant metastases from papillary and follicular thyroid carcinoma: Benefits and limits of radioiodine therapy. J. Clin. Endocrinol. Metab..

[2] Fugazzola L., Elisei R., Fuhrer D., Jarzab B., Leboulleux S., Newbold K., Smit J. (2019). 2019 European Thyroid Association Guidelines for the Treatment and Follow-Up of Advanced Radioiodine-Refractory Thyroid Cancer. Eur. Thyroid. J..

[3] Chougnet C., Brassard M., Leboulleux S., Baudin E., Schlumberger M. (2010). Molecular targeted therapies for patients with refractory thyroid cancer. Clin. Oncol. (R. Coll. Radiol.).

[4] Araque K.A., Gubbi S., Klubo-Gwiezdzinska J. (2020). Updates on the Management of Thyroid Cancer. Horm. Metab. Res..

[5] Wells S.A., Robinson B.G., Gagel R.F., Dralle H., Fagin J.A., Santoro M., Baudin E., Elisei R., Jarzab B., Vasselli J.R. (2012). Vandetanib in patients with locally advanced or metastatic medullary thyroid cancer: A randomized, double-blind phase III trial. J. Clin. Oncol..

[6] Elisei R., Schlumberger M., Müller S.P., Schöffski P., Brose M.S., Shah M.H., Licitra L., Jarzab B., Medvedev V., Kreissl M.C. (2013). Cabozantinib in progressive medullary thyroid cancer. J. Clin. Oncol..

[7] Brose M.S., Nutting C.M., Jarzab B., Elisei R., Siena S., Bastholt L., Fouchardiere C.d.l., Pacini F., Paschke R., KeeShong Y. (2014). Sorafenib in radioactive iodine-refractory, locally advanced or metastatic differentiated thyroid cancer: A randomised, double-blind, phase 3 trial. Lancet.

[8] Schlumberger M., Tahara M., Wirth L.J., Robinson B., Brose M.S., Elisei R., Habra M.A., Newbold K., Shah M.H., Hoff A.O. (2015). Lenvatinib versus placebo in radioiodine-refractory thyroid cancer. N. Engl. J. Med..

[9] Lorusso L., Pieruzzi L., Biagini A., Sabini E., Valerio L., Giani C., Passannanti P., Pontillo-Contillo B., Battaglia V., Mazzeo S. (2016). Lenvatinib and other tyrosine kinase inhibitors for the treatment of radioiodine refractory, advanced, and progressive thyroid cancer. OncoTargets Ther..

[10] Kirtane K., Roth M.Y. (2020). Emerging Therapies for Radioactive Iodine Refractory Thyroid Cancer. Curr. Treat. Options Oncol..

[11] Wen T., Xiao H., Luo C., Huang L., Xiong M. (2017). Efficacy of sequential therapies with sorafenib-sunitinib versus sunitinib-sorafenib in metastatic renal cell carcinoma: A systematic review and meta-analysis. Oncotarget.

[12] Hiraoka A., Kumada T., Atsukawa M., Hirooka M., Tsuji K., Ishikawa T., Takaguchi K., Kariyama K., Itobayashi E., Tajiri K. (2019). Real-life Practice Experts for HCC (RELPEC) Study Group; HCC 48 Group (hepatocellular carcinoma experts from 48 clinics in Japan). Important Clinical Factors in Sequential Therapy Including Lenvatinib against Unresectable Hepatocellular Carcinoma. Oncology.

[13] Park K., Bennouna J., Boyer M., Hida T., Hirsh V., Kato T., Lu S., Mok T., Nakagawa K., O’Byrne K. (2019). Sequencing of therapy following first-line afatinib in patients with EGFR mutation-positive non-small cell lung cancer. Lung Cancer.

[14] Kurzrock R., Sherman S.I., Ball D.W., Forastiere A.A., Cohen R.B., Mehra R., Pfister D.G., Cohen E.E.W., Janisch L., Nauling F. (2011). Activity of XL184 (Cabozantinib), an oral tyrosine kinase inhibitor, in patients with medullary thyroid cancer. J. Clin. Oncol..

[15] Cabanillas M.E., Brose M.S., Holland J., Ferguson K.C., Sherman S.I. (2014). A Phase I Study of Cabozantinib (XL184) in Patients with Differentiated Thyroid Cancer. Thyroid.

[16] Cabanillas M.E., Schlumberger M., Jarzab B., Martins R.G., Pacini F., Robinson B., McCaffrey J.C., Shah M.H., Bodenner D.L., Topliss D. (2015). A phase 2 trial of lenvatinib (E7080) in advanced, progressive, radioiodine-refractory, differentiated thyroid cancer: A clinical outcomes and biomarker assessment. Cancer.

[17] Bible K.C., Suman V.J., Molina J.R., Smallridge R.C., Maples W.J., Menefee M.E., Rubin J., Karlin N., Sideras K., Morris J.C. (2014). A Multicenter Phase 2 Trial of Pazopanib in Metastatic and Progressive Medullary Thyroid Carcinoma: MC057H. J. Clin. Endocrinol. Metab..

[18] Atallah V., Hocquelet A., Do Cao C., Zerdoud S., De La Fouchardiere C., Bardet S., Italiano A., Dierick-Galet A., Leduc N., Bonichon F. (2016). Activity and Safety of Sunitinib in Patients with Advanced Radioiodine Refractory Thyroid Carcinoma: A Retrospective Analysis of 57 Patients. Thyroid.

[19] Dadu R., Devine C., Hernandez M., Waguespack S.G., Busaidy N.L., Hu M.I., Jimenez C., Habra M.A., Sellin R.V., Ying A.K. (2014). Role of salvage therapy in differentiated thyroid cancer patients who failed first-line sorafenib. J. Clin. Endocrinol. Metab..

[20] Cabanillas M.E., de Souza J.A., Geyer S., Wirth L.J., Menefee M.E., Liu S.V., Shah K., Wright J., Shah M.H. (2017). Cabozantinib As Salvage Therapy for Patients with Tyrosine Kinase Inhibitor-Refractory Differentiated Thyroid Cancer: Results of a Multicenter Phase II International Thyroid Oncology Group Trial. J. Clin. Oncol..

[21] Massicotte M.H., Brassard M., Claude-Desroches M., Borget I., Bonichon F., Giraudet A.-L., Cao C.D., Chougnet C.N., Sophie Leboulleux S., Eric Baudin E. (2014). Tyrosine kinase inhibitor treatments in patients with metastatic thyroid carcinomas: A retrospective study of the TUTHYREF network. Eur. J. Endocrinol..

[22] Brose M.S., Cabanillas M.E., Cohen E.E., Wirth L.J., Riehl T., Yue H., Sherman S.I., Sherman E.J. (2016). Vemurafenib in patients with BRAF(V600E)-positive metastatic or unresectable papillary thyroid cancer refractory to radioactive iodine: A non-randomised, multicentre, open-label, phase 2 trial. Lancet Oncol..

[23] Oh H.S., Shin D.Y., Kim M., Park S.Y., Kim T.H., Kim B.H., Kim E.Y., Kim W.B., Chung J.H., Shong Y.K. (2019). Extended Real-World Observation of Patients Treated with Sorafenib for Radioactive Iodine-Refractory Differentiated Thyroid Carcinoma and Impact of Lenvatinib Salvage Treatment: A Korean Multicenter Study. Thyroid.

[24] Eisenhauer E.A., Therasse P., Bogaerts J., Schwartz L.H., Sargent D., Ford R., Dancey J., Arbuck S., Gwyther S., M Mooney M. (2009). New response evaluation criteria in solid tumours: Revised RECIST guideline (version 1.1). Eur. J. Cancer.

[25] Owonikoko T.K., Chowdry R.P., Chen Z., Kim S., Saba N.F., Shin D.M., Khuri F.R. (2013). Clinical efficacy of targeted biologic agents as second-line therapy of advanced thyroid cancer. Oncologist.

[26] Shi R.L., Qu N., Liao T., Wei W.J., Wang Y.L., Ji Q.H. (2016). The Trend of Age-Group Effect on Prognosis in Differentiated Thyroid Cancer. Sci. Rep..

[27] Kauffmann R.M., Hamner J.B., Ituarte P.H.G., Yim J.H. (2018). Age greater than 60 years portends a worse prognosis in patients with papillary thyroid cancer: Should there be three age categories for staging?. BMC Cancer.

[28] Brose M.S., Worden F.P., Newbold K.L., Guo M. (2017). Effect of Age on the Efficacy and Safety of Lenvatinib in Radioiodine-Refractory Differentiated Thyroid Cancer in the Phase III SELECT Trial. J. Clin. Oncol..

[29] Locati L., Piovesan A., Durante C., Bregni M., Castagna M.G., Zovato6 S., Giusti M., Ibrahim T., Puxeddu E., Fedele G. (2019). Real-world efficacy and safety of lenvatinib: Data from a compassionate use in the treatment of radioactive iodine-refractory differentiated thyroid cancer patients in Italy. Eur. J. Cancer.

[30] Cantara S., Bertelli E., Occhini R., Regoli M., Brilli L., Pacini F., Castagna M.G., Toti P. (2019). Blockade of the programmed death ligand 1 (PD-L1) as potential therapy for anaplastic thyroid cancer. Endocrine.

[31] French J.D. (2020). Immunotherapy for advanced thyroid cancers—Rationale, current advances and future strategies. Nat. Rev. Endocrinol..

